# Homœopathic Colleges

**Published:** 1884-09

**Authors:** Alice B. McKibben


### HOMŒOPATHIC COLLEGES.

In the August number of THE HOMŒOPATHIC PHYSICIAN the first article which attracts attention is one on Medical Education, by Dr. P. P. Wells. After carefully reading the said article, the question immediately suggests itself: In what does a medical college differ from other colleges and from other institutions of special training? Considering all the points made by the author in favor of longer time, the number of branches taught, greater efficiency in the teachers, together with more proficient students, we find the same general charges that are made in common with all institutions; but when he says that "the very knowledge the students came for, has been withheld from them by the negligence or incompetence of those whose duty it was to teach the philosophy as well as the practice of our therapeutics," then there is a definite and specific charge against homœopathic medical colleges, and it behooves every earnest Hahnemannian to be up and about as an investigating committee with power to institute a reform of methods of teaching. The *Organon* is the basis of all true homœopathic teaching, and if this is neglected by any college professing to be homœopathic, and as such graduating men and women as homœopathic physicians, there need be but little difficulty in assigning such colleges to their true position. For certainly their students are not taught according to the principles of either allopathy or homœopathy. What would be said of Columbia College, or any other institution, whose basis was the teaching of the subject of the law, if Blackstone and Coke, the Magna Charta, or any of the great and well-recognized and received writings in which the philosophy and generalizations of the law are stated were not taught? Does any one believe that a graduate of any theological college, however well versed in the sciences of the world, the literature of ages, the history of nations, or the arts of civilization, but ignorant of the Bible, could, by an exhibition of a diploma, by any statement of self or others, be accepted as a minister of the Gospel or expounder of truth by any special sect of believers? The demand of a minister is that he shall *know* the law that governs him. The demand of the lawyer is that he shall *know* the nature of the law of the land, both specially and generally. What makes us a distinctive school of medicine is our own perfect law of cure and certainly those institutions that fall so far short of the "mark of their high calling" can in no sense be called "our best" colleges. Dr. Wells says: "And yet students but recently from the class-room, at the close of the term of two of our best (!!!) colleges, have assured me they have heard no word of the philosophy of this science from the first to the last of the course."

It has been a habit to look to the East as the source of all the best methods of teaching in medicine, as we look for the first strong, bright rays of the sunlight, but after such a statement from the great medical centre, we realize again that "Westward the star of the empire takes its way," for in the Homœopathic College of Missouri the *Organon is the basis of instruction*, and so thoroughly is it taught, that the highest test may be applied, namely, the desire of those who have been pupils there is to drink deep of this "Pierian Spring."

ALICE B. MCKIBBEN, M. D.

NOTE.—THE HOMŒOPATHIC PHYSICIAN is glad to print Dr. McKibben's letter, calling attention to the method of teaching Homœopathy pursued by the faculty of the Homœopathic Medical College of Missouri. It is time that homœopathic physicians should understand definitely that some of our so-called colleges *do not* teach Homœopathy and should make a distinction in their recommendations of these schools.

Homœopathy cannot be properly taught in any school that does not require a *thorough study* of the *Organon*. We have three or more good schools; the others are worthless and should be "boycotted."—EDITOR.

